# Willingness, Self-Perceived Barriers, and Practices of Pharmacists Toward Extended Pharmacy Services for Health Promotion: A Cross-Sectional Survey in Karachi, Pakistan †

**DOI:** 10.3390/pharmacy14030079

**Published:** 2026-05-28

**Authors:** Sadia Shakeel, Hina Rehman, Shagufta Nesar, Muskan Bhutto, Imran Ahsan Mallick, Márió Gajdács, Shazia Jamshed

**Affiliations:** 1 Department of Pharmacy Practice, Faculty of Pharmaceutical Sciences, Dow College of Pharmacy, Dow University of Health Sciences, Karachi 74200, Pakistan; imran.ahsan@duhs.edu.pk; 2 Department of Pharmacy Practice, Institute of Pharmaceutical Sciences, Jinnah Sindh Medical University, Karachi 75510, Pakistan; hina.rehman@jsmu.edu.pk (H.R.); muskanbhutto948@gmail.com (M.B.); 3 Jinnah College of Pharmacy, Sohail University, Karachi 75900, Pakistan; shaguftausmani@sohailuniversity.edu.pk; 4 Department of Public Health, Albert Szent-Györgyi Medical School, University of Szeged, 6720 Szeged, Hungary; 5 Department of Pharmacy Practice, School of Pharmacy, International Medical University (IMU), Kuala Lumpur 57000, Malaysia; shaziajamshed@imu.edu.my; 6 Department of Pharmacy Practice, School of Pharmaceutical Sciences, Shifa Tameer-e-Millat University, Islamabad 44000, Pakistan; 7 Department of Pharmacy Practice, Faculty of Pharmacy, Jinnah University for Women, Karachi 74600, Pakistan

**Keywords:** pharmacists, health promotion, community pharmacy service, knowledge, attitudes, practices, primary healthcare, expanded services, public health, Pakistan

## Abstract

**Background/Objectives**: Health promotion (HPr) is increasingly recognized as an essential component of modern pharmacy practice. In developing countries like Pakistan, pharmacists’ roles are evolving from traditional dispensing toward extended pharmacy services (EPS). This study evaluated the willingness, knowledge, attitudes, and perceived barriers of pharmacists in Karachi, Pakistan, regarding the provision of HPr-focused EPS. **Methods**: An online, cross-sectional, observational study was conducted between October 2024 and April 2025, using a 32-item questionnaire. Purposive and snowball sampling were employed to recruit pharmacists, including interns and fresh graduates. Descriptive and inferential statistics (χ^2^, Fisher’s exact and Welch’s *t*-tests) were used for data analysis. **Results**: Of *N* = 389 respondents (mean age 29.8 ± 4.8 years), 85.1% expressed willingness to provide HPr services. A majority of respondents (72.7% and 70.4%, respectively) felt they possessed sufficient knowledge for HPr and for identifying lifestyle-related risks, while more experienced pharmacists (>1 year) reported higher confidence (*p* < 0.001 and *p* = 0.043). Positive attitudes toward public health involvement were high (82.3%), particularly among females (*p* < 0.001), younger pharmacists and fresh graduates (*p* = 0.019 and *p* = 0.010). However, only 39.1% believed they had sufficient time for patient education. Practicing pharmacists (*n* = 114) were most frequently involved in dietary advice (4.38 ± 0.89) and medication safety education (4.36 ± 0.97), while family planning counseling (2.92 ± 1.36) was the least commonly reported activity. Leading barriers identified via word cloud analysis included lack of time (*n* = 101), perceived lack of necessary skills (*n* = 96), insufficient resources (*n* = 91), limited technology access (*n* = 89), and lack of specific protocols (*n* = 84). **Conclusions**: Pakistani pharmacists demonstrate high professional willingness to engage in HPr-focused EPS. However, systemic barriers—primarily time constraints and a lack of supportive infrastructure—impede the full integration of these services into routine practice. Policy interventions, standardized protocols, and reimbursement models are necessary to leverage pharmacists’ potential in primary healthcare.

## 1. Introduction

Health promotion (HPr) is a multidisciplinary process, enabling individuals and communities to enhance control over their health determinants and to improve overall health outcomes through education, empowerment, and health-supportive environments [1]. HPr utilizes a range of complementary strategies to address different health issues prevailing in society, including health communication and education, regulatory perspectives, financial incentives, organizational reforms, community development and cohesion, and spontaneous local events [2]. Principally, HPr directs activities aimed at enhancing the health and well-being both at individual and community levels. Due to the transformation of pharmacy practice globally, HPr is increasingly acknowledged as an essential part of modern, patient-facing pharmacy practice [3]. Pharmacists’ expertise in the procurement, dispensing and management of medications may have a direct impact on public health. Through HPr activities, they may enhance patient outcomes and reduce healthcare expenditures. Global health authorities, including the World Health Organization (WHO) and the International Pharmaceutical Federation (FIP), emphasize the expanding role of pharmacists in primary healthcare and preventive services, especially within the framework of task-shifting [4]. Being among the most accessible healthcare professionals, pharmacists often serve as the first point of contact within the healthcare system. In addition, pharmacists contribute to clinical–community linkages by identifying social determinants of health that may influence a person’s health status, increasingly being involved in implementing disease prevention strategies through immunization, early detection of certain conditions, symptom management, and chronic disease counseling and care [5]. Furthermore, they may play an important role in patient education, self-care support, facilitating medication adherence, referral guidance, and the promotion of rational use of medicines and resources of the healthcare system [6].

The evolution of pharmacy practice in developing countries, such as Pakistan, is constrained by specific challenges with regard to professional recognition, a clear and delineated position within the country’s healthcare infrastructure, perspectives on education and training, and regulatory frameworks [7]. Pakistan’s healthcare system, which is characterized by a complex blend of public and private healthcare service providers, has major challenges in delivering high-quality and equitable services to its population of around ~258 million persons [8]. Primary healthcare services remain under-resourced, and community pharmacies are often the most accessible point of contact for patients, particularly in urban and peri-urban settings. However, pharmacy practice in Pakistan remains largely product-oriented, with limited integration of pharmacists into structured clinical and HPr-associated roles. Variability in training exposure, regulatory enforcement, and professional recognition further contributes to inconsistent implementation of patient-centered services, including HPr activities. Hence, the profession in the country lags behind as compared to developed countries, where—depending on the levels of service integration and task-shifting—pharmacists play important roles in a variety of contexts, including chronic illness management, offering patient counseling, delivering immunizations, and even prescribing for certain indications, as a part of extended pharmacy services (EPS) [9]. However, regional comparisons reveal similarities with other developing countries, such as Bangladesh and India, where community pharmacies rarely offer services associated with HPr and prevention, preferring to focus on the conventional (i.e., curative/therapeutic) tasks of dispensing medications [5]. Limited empirical evidence exists locally regarding pharmacists’ knowledge, attitudes, and practices (KAP) in HPr activities across diverse settings. In particular, little is known about their readiness to provide EPS, and the barriers associated with their implementation into everyday routine practice. This lack of comprehensive data hinders the effective elimination of the persisting roadblocks, and the successful integration of pharmacists into structured public health programs. Hence, the current study was carried out to evaluate the degree of pharmacists’ willingness and perceptions towards providing EPS, in particular pharmacist-delivered HPr services, and the perceived barriers that could limit their participation across diverse settings. The findings of the present study may aid the closure of the gap in the literature, and provide policymakers, public health agencies, and other healthcare professionals with crucial information pertaining to the perceived readiness and obstacles of pharmacists to provide HPr services.

## 2. Materials and Methods

### 2.1. Study Design and Duration

An online questionnaire-based, cross-sectional, observational study was performed between October 2024 and April 2025. A non-probabilistic sampling approach (i.e., purposive and snowball sampling) was applied, due to the wide distribution of pharmacists across different healthcare settings and the lack of a comprehensive accessible and comprehensive sampling list, to include members of our target population (i.e., pharmacists in Karachi, Pakistan) willing to participate in the study during the time of the data collection.

The study adheres to the STROBE (Strengthening the Reporting of Observational Studies in Epidemiology) guidelines for cross-sectional studies to ensure methodological rigor, transparency, and reproducibility [10]; the STROBE checklist is provided in Supplementary Material S1.

### 2.2. Ethical Considerations

The study was conducted in accordance with the World Medical Association (WMA) Declaration of Helsinki (1975, last revised in 2024), and national and institutional ethical standards. Ethical approval for this study was obtained from the Ethical Review Committee of Sohail University, Karachi (approval ID: 00066/24; date of the approval: 27 September 2024).

Before participation in our study, pharmacists were provided with a detailed description of the study’s goals, procedures, potential risks, and benefits. The participant information sheet was embedded at the beginning of the online questionnaire, and access to the survey was only permitted after participants provided electronic informed consent, ensuring that they had read and understood the study information prior to participation. They were informed that their responses will solely be used for research purposes, their participation is voluntarily, and that they have the right to withdraw at any time without any negative consequences. Confidentiality and anonymity was maintained throughout the study. The participants did not receive any incentives (monetary or otherwise) to take part in the study.

### 2.3. Study Population, Inclusion and Exclusion Criteria, Sample Size Determination

Data collection was performed among pharmacists working in the public and private healthcare sector in Karachi, Pakistan. The inclusion criteria of the study were as follows: (i) pharmacists who were registered with the Pharmacy Council of Sindh (including community pharmacists, hospital pharmacists, fresh graduates and intern pharmacists), (ii) willing to take part in the study voluntarily, and provided written informed consent, (iii) able to read and comprehend English, allowing for the filling-out of the data collection instrument, (iv) who completed the entire questionnaire.

The exclusion criteria of the study were: (i) healthcare and allied health staff, not holding the above-mentioned certifications, (ii) those who were unable to comprehend English to a sufficient degree to fill out the questionnaire, (iii) pharmacists refusing to take part in the study (due to refusal of providing informed consent), (iv) those who submitted a questionnaire with incomplete responses.

Based on the estimations of the FIP Global Pharmaceutical Observatory, there are between 55,000 and 63,875 registered pharmacists in Pakistan, with ~12,000 being in the Sindh region. The minimum required sample size for the study was determined using the Raosoft Sample Size Calculator (https://raosoftcalculator.com/; accessed on 1 May 2024), using the formula described below (1):

(1) $$n=N\frac{x}{\left(N-1\right)E2+x}$$

where “*x*” is the expected response rate, “*E*” is the acceptable margin of error (5%, i.e., the required level of confidence was 95%), the population (*N*) was set at 12,000 (based on data described above), and the expected response rate was set at 50% [11]. The required sample size of *N* = 271 was set for the completion of this study.

### 2.4. Development of the Questionnaire, Data Collection

The instrument for data collection was a self-administered, structured, online questionnaire, which was developed for the purposes of the study, after conducting a comprehensive literature review (with studies published up until 2023) of similar studies, and identifying relevant papers based on the study’s geographical and health system context [3,12,13,14]. First, a preliminary version of the questionnaire was developed, and an expert panel (including researchers in pharmacy practice research, community pharmacists, an epidemiologist and a senior academic) assessed the questionnaire for its face and content validity. The questionnaire was designed using closed-ended questions and Likert scales to allow uniform responses, facilitate analysis, and maintain confidentiality. After the modifications in the questionnaire were adopted, based on the comments of the panel, the final survey tool was distributed among participants.

The questionnaire consisted of 32 items, with five main sections, as follows: (i) socio-demographic and professional characteristics (pharmacy practice setting, working experience and willingness to provide pharmacist-delivered HPr services), (ii) knowledge-assessment domain (5 items), including questions assessing participants’ awareness and understanding of extended pharmacy services to HPr and prevention (possible responses: strongly disagree → strongly agree), (iii) attitude-assessment domain (8 items), assessing their perceptions, beliefs, readiness to participate in public health and HPr activities (possible responses: strongly disagree → strongly agree), (iv) practice-assessment domain (11 items), evaluating participants’ actual involvement in counseling, research initiatives, and screening services (possible responses: never → always). Fresh graduates and intern pharmacists were not eligible to fill out the practice-assessment domain, as these items required real-world professional experience. For intern pharmacists/fresh graduates, practice items included a “Not applicable/I have not performed this activity” option to ensure item relevance, (v) self-perceived barriers: the respondents were asked to select all applicable barriers perceived to provide pharmacist-delivered HPr services (multiple choice, with an open-ended “Other” option), which were visualized in the form of a word cloud. Fresh graduates and intern pharmacists were not eligible to fill out the section on self-perceived barriers. The link to access the final questionnaire was shared on various social media platforms to reach the intended target population.

### 2.5. Statistical Analysis

Data were collected using an online survey platform. Responses were automatically recorded and exported to Microsoft Excel (Microsoft Corp., Redmond, WA, USA), and subsequently transferred to Statistical Package for Social Sciences 27.0 (IBM Corp., Endicott, NY, USA) for analysis. Prior to analysis, data were screened for completeness and consistency, and incomplete responses were excluded to ensure data quality and integrity. During descriptive analysis, categorical data were described as frequencies and percentages (*n*, %), while continuous data was described as mean ± standard deviation [SD]. For continuous variables, normality testing was performed using Kolmogorov–Smirnov tests and Q-Q (quantile-quantile) diagrams. Associations between categorical variables were assessed using χ^2^-tests and Fisher’s exact tests, with Cramér’s V effect size measure [φ], in case of significant results. During comparison of continuous variables, Welch’s *t*-tests were performed. *p*-values below 0.05 (*p* < 0.05) were considered statistically significant. Due to the previously detailed exclusion criteria, missing data were not relevant during analyses.

A word cloud was generated using the online platform WordClouds.com (https://www.wordclouds.com/; accessed on 1 May 2024) to visualize perceived barriers reported by participants. Multi-word phrases were retained as complete phrases to preserve semantic meaning, and phrase size reflected the relative frequency of each reported barrier to participate in HPr services.

### 2.6. Reporting Guidelines

This manuscript adheres to the Strengthening the Reporting of Observational Studies in Epidemiology (STROBE) guidelines for cross-sectional studies; the STROBE checklist is provided in Supplementary Material S1.

## 3. Results

### 3.1. Socio-Demographic and Professional Characteristics of the Participants

During data collection, out of the 493 individuals who started filling out the survey, a total of *N* = 389 (100.0%) respondents provided complete questionnaires, which were included in the final analysis. Due to the online distribution of the survey through multiple social media platforms, the exact number of pharmacists who were approached with the questionnaire could not be determined, and therefore the response rate could not be accurately calculated.

The main socio-demographic and professional characteristics of our sample are shown in Table 1. The mean age of participants was 29.8 ± 4.8 years, with 61.4% (*n* = 239) being ≤30 years of age and female (*n* = 217, 55.8%). Regarding their practice setting, most participants were pharmacy interns or fresh graduates (*n* = 275, 70.7%; resulting in 0–5 years of working experience in 80.7% of respondents), followed by hospital pharmacists (*n* = 69, 17.7%) and community pharmacists (*n* = 45, 11.6%), respectively; more than two-thirds (*n* = 269, 69.1%) worked in the private sector. Most participants stated being willing to provide pharmacists-based HPr services (*n* = 331, 85.1%) (Table 1).

### 3.2. Pharmacists’ Knowledge Regarding HPr and Disease Prevention

The results observed based on the knowledge-assessment domain in our questionnaire are shown in Table 2. Overall, 72.7% (*n* = 283) of participants agreed or strongly agreed that they had sufficient knowledge to advise patients on HPr activities and disease prevention; those who were not pharmacy interns, >30 years of age and with >1 year of experience were more likely to agree (*p* = 0.036, *p* < 0.001 and *p* < 0.001, respectively). In total, 66.9% (*n* = 260) of participants agreed or strongly agreed that they can provide health education beyond issues related to medicines; female participants who were not pharmacy interns were more likely to agree (*p* = 0.012 and *p* = 0.004). Confidence in identifying patients at risk for lifestyle-related diseases was reported by 51.4% (*n* = 200) participants who agreed and 19.0% (*n* = 74) who strongly agreed; those with ≥1 year of experience were more likely to do so (*p* = 0.043). The perceived adequate knowledge to counsel on diet and nutrition was reported by 48.8% (*n* = 190) participants who agreed and 10.5% (*n* = 41) who strongly agreed; those who were not pharmacy interns, >30 years of age and with >1 year of experience were more likely to agree (*p* = 0.017, *p* = 0.011 and *p* = 0.004, respectively). Self-perceived adequate knowledge on the preventive measures for common chronic diseases was agreed upon by 45.0% (*n* = 175) and strongly agreed upon by 9.5% (*n* = 37) of participants; those >30 years of age and with >1 year of experience were more likely to agree (*p* = 0.013 and *p* = 0.035).

### 3.3. Participants’ Attitudes Towards Involvement in Public Health Activities

The results observed based on the attitude-assessment domain in our questionnaire are shown in Table 3. Most agreed or strongly agreed that professional curricular training during their undergraduate education was adequate (67.1%, *n* = 261); fresh graduates, those ≤30 years of age, and <1 years of experience were more likely to agree (*p* = 0.002, *p* < 0.001 and *p* < 0.001, respectively). A total of 82.3% (*n* = 320) of participants agreed or strongly agreed that pharmacists should actively participate in public health activities; females (*p* < 0.001), those with ≤30 years of age, and <1 years of experience were more likely to agree (*p* = 0.019 and *p* = 0.010). The majority recognized pharmacists’ important role in HPr, alongside physicians and nurses (87.9%; *n* = 339); those who were not pharmacy interns were more likely to agree (*p* = 0.042). Only 39.1% (*n* = 152) believed that they have sufficient time to educate their patients on health issues during pharmacist–patient encounters: those who were not pharmacy interns, those working in private settings, >30 years of age and with >1 year of experience were less likely to agree (*p* = 0.013, *p* = 0.003, *p* < 0.001 and *p* < 0.001, respectively). A total of 38.5% (*n* = 150) of participants concurred that collaboration with other health workers allows them to perform public health functions more effectively, with those working in the public sector more likely to do so (*p* < 0.001). In total, 80.4% (*n* = 313) felt ready to engage in public health activities, with fresh graduates more likely to agree (*p* < 0.001). Some participants, 36.7% (*n* = 143), agreed that community pharmacies may be suitable places to conduct public health activities; those with ≤30 years of age, and <1 years of experience were more likely to agree (*p* = 0.010 and *p* = 0.005, respectively). Finally, 51.4% (*n* = 200) agreed or strongly agreed that people will accept and appreciate their involvement in public health activities; fresh graduates, those with ≤30 years of age, and <1 years of experience were more likely to agree (*p* = 0.016, *p* = 0.035 and *p* = 0.025, respectively) (Table 3).

### 3.4. Participants’ Involvement in Public Health Activities, Perceived Barriers

Out of a total of *N* = 389 (100.0%) respondents, only *n* = 114 (community and hospital pharmacists) were eligible to take part in the final domains of the questionnaire, while intern pharmacists were excluded from this section. The frequency of their involvement in public health activities is shown in Table 4. Instructions on following a healthy diet were most commonly reported (4.38 ± 0.89), followed by providing education on the appropriate practices for disposing expired/unusable drugs (4.36 ± 0.97), where most respondents reported “always” providing these services (62.3% and 65.8%, respectively). Chronic disease counseling (4.05 ± 1.01), education on the use of medical instruments (3.96 ± 1.23), education on appropriate oral hygiene (3.91 ± 1.10) and weight management counseling (3.88 ± 1.03) were moderately common. In contrast, lower levels of engagement were reported for smoking cessation counseling (3.74 ± 1.42), lifestyle modification counseling (3.72 ± 1.15), and counseling on traditional medicine practices (3.77 ± 1.22), despite around half of respondents reporting “always” performing smoking cessation education (50.9%). The lowest engagement was noted for counseling for family planning (2.92 ± 1.36), where only 21.9% reported “always” providing the service. With the exception of female pharmacists more commonly involved in family planning (3.45 ± 0.98 vs. 2.17 ± 1.47; *p* = 0.032) and smoking cessation counseling (3.96 ± 1.12 vs. 3.28 ± 0.67; *p* = 0.045), no relevant differences were observed based on a participant’s gender, working experience or organizational setting (*p* > 0.05).

**Figure 1:**
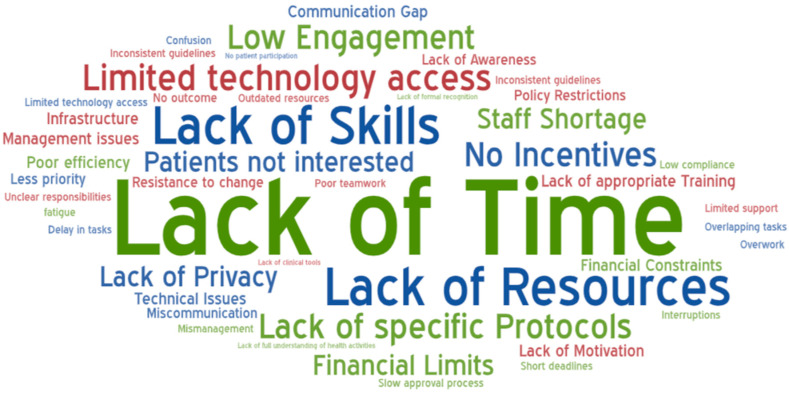
Word cloud analysis—based on frequency of word occurrence—highlighting the main barriers identified to participate in EPS by practicing pharmacists.

The word cloud analysis highlighted the main barriers identified by participants, (Figure 1). Word size represents the relative frequency of the barriers. Lack of time emerged as the most frequently mentioned barrier, reported by *n* = 101 participants, followed closely by the perceived lack of necessary skills (*n* = 96), lack of resources (*n* = 91), limited access to technology (*n* = 89), lack of specific protocols (*n* = 84), low engagement (*n* = 82), and lack of incentives (*n* = 75), while other notable barriers included lack of patients’ interest (*n* = 50), lack of privacy to perform counseling (*n* = 45), financial constraints (*n* = 40) and staff shortage (*n* = 38), respectively.

## 4. Discussion

Over the past twenty years, the roles of pharmacists have undergone a paradigm shift, from a focus that was product or medicine-oriented (i.e., dispensing), to a patient-centric model of care, within the framework of EPS. Studies have reported a progressive shift in patient perceptions toward pharmacist-led advanced clinical and preventive services, particularly in developed countries [1,4]. Following the COVID-19 epidemic, pharmacists’ responsibilities have grown notably, and they now play more pronounced roles in the healthcare system than distributing prescription drugs only [15,16,17,18,19,20]. Pharmacists may serve as the healthcare liaison that improves access to and equity of healthcare by bringing patients closer to their physicians. Patients consider pharmacists capable of providing a wide range of services beyond traditional dispensing roles, including medication therapy management, vaccination services, chronic disease screening, and HPr interventions. In many low- and middle-income countries (LMICs), including Pakistan, pharmacies are often the most accessible healthcare settings for the general population within an overburdened healthcare system. However, evidence from LMICs indicates that these services are being adopted at a slower pace [7]. This is mostly because of lack of public awareness, inadequate clinical pharmacy infrastructure, and the conventional belief that pharmacists are only responsible for dispensing medications. The current study assessed Pakistani pharmacists’ willingness, insights and perceived barriers to provide EPS—focusing on HPr services—to provide a benchmark on the opinions of the pharmacy workforce, and to potentially identify the systemic barriers that impede the transition of pharmacists from offering “traditional” pharmacy services to EPS. The demographic profile of participants in the present study reflects a young and early-career pharmacy workforce, with a majority of pharmacy interns and fresh graduates. Pharmacy interns/fresh graduates were included as they undergo structured clinical training in both hospital and community settings, which provides them with exposure to HPr and patient counseling activities. Their inclusion in the study allows assessment of both current practice and early professional readiness, which is principally attributable to the quality of training provided by undergraduate programs throughout Pakistan. More than half of the respondents were female, aligning with the growing feminization trend observed in the pharmacy profession in Pakistan and other developing countries [7,15].

Encouragingly, a vast majority (>85%) expressed willingness to provide HPr services, highlighting the awareness towards this extended professional responsibility among our participants. High levels of willingness in the current study to engage in HPr services aligns with earlier global reports, emphasizing the expanding role of pharmacists in preventive care (especially in urban environments) [1], and the willingness of community pharmacists to offer HPr within the framework of EPS [16,17]. In contrast, the recent study of Jarab et al. from the United Arab Emirates has highlighted unsatisfactory levels of attitude and low willingness towards participating in EPS among its respondents, especially among women—and has identified several barriers providing additional services (including a lack of clinical problem-solving skills, lack of specific, HPr or EPS-related training and the absence of a private consultation room for HPr) [12]. Female pharmacists typically take on additional caregiving responsibilities at home, and may have less time or inclination to undertake HPr, as it could impact their work–life balance, leading to adverse opinions on EPS [12]. In addition, the study of Korayem et al. reported that pharmacists who were PharmD graduates reported a greater willingness to deliver HPr services than BPharm holders; this phenomenon may be explained by the fact that PharmD graduates usually receive more in-depth training in clinical pharmacy practice, patient counseling, and medication management, thereby placing a greater emphasis on patient-centered care and inter-professional collaboration, which could be the reason for the increased willingness to provide EPS and HPr [18].

Almost two-thirds of respondents were confident in their knowledge to provide HPr and disease-prevention advice, and over half of them felt confident on counseling on nutrition, and in identifying patients who are at risk of developing lifestyle-related diseases; overall, more experienced pharmacists were more comfortable with their HPr-related competencies. Likewise, an earlier Pakistani study by Kamal et al. reported that participants showed sufficient knowledge regarding the evolution of their practice, with 45.5% reporting significant improvements in community pharmacy practice and 47.4% reporting a modest rise in service offerings [19]. On the other hand, as a general rule, fresh graduates and younger participants presented with more positive attitudes, suggesting that their undergraduate education sufficiently prepared them to undertake HPr activities, they recognized the role of pharmacists in HPr, and they felt engaged in public health activities, as well as were aware of the importance of inter-professional collaboration, especially by those working in the public sector. In contrast, according to Palaian et al., pharmacists agreed that taking on EPS would require clinical knowledge upskilling [16]. Likewise, the majority (>75.0%) of participants in the study of Jarab et al. believed that extended pharmacy services require considerable upskilling in clinical knowledge (77.7%), and noting that pharmacy education never equipped them with the necessary abilities [12]; the perceived lack of necessary skills, and the lack of guidelines were also noted by our participants. Despite the fact that an earlier study from Pakistan showed that pharmacists were ignorant of pharmaceutical care, they were receptive to a change in practice if provided with the necessary skills and training [20].

Consistently, the vast majority of the participants recognized that pharmacists have an important role in public health alongside other healthcare professionals. However, less than 40.0% believed that pharmacist–patient encounters allow for HPr-related activities (highlighting both infrastructural and time constraints, especially among more experienced pharmacists, working in privately owned facilities). Our results showcased an additional perspective, i.e., only around half of respondents believed that their patients would be willing to receive HPr and other EPS-related services from them. Low engagement and lack of interest from the patient’s side were also reported as notable limitations by our participants. Our findings are in line with earlier results published by Nsengimana et al., demonstrating that community pharmacies’ inability to provide HPr and preventive services was caused by consumers’ lack of time to engage with the pharmacist [13]. This is discordant with global trends, in which dispensing is still a fundamental pharmacy role, but it also points to a slow advancement toward the more advanced therapeutic services observed in countries of the Global North. The study of El-Kholy et al. reported that 70.2% of the public trusted pharmacists’ opinions regarding medications, and 72.8% thought that pharmacists gave them clear instructions about how to use medications. In addition, around two-thirds of respondents stated that they were satisfied with services provided by pharmacy staff [21]. Furthermore, Alanazi et al. reported that the majority (61.0%) of pharmacy customers in Pakistan trusted pharmacists, and were satisfied with how the pharmacists addressed issues [22]. Having limited time to educate patients on health issues was brought up by participants in the study of Sendekie et al., where they concluded that community pharmacists would be more inclined toward HPr activities, if they had to deal with fewer patients of a daily basis, freeing up time from administrative and dispensing tasks to advance more EPSs [3].

All healthcare professionals (especially pharmacists and physicians) need to embrace the concept of inter-professional collaborative teamwork, to achieve the best possible outcomes for the population through multidisciplinary efforts. Francisco et al. proposed a model that highlights the transformative potential of pharmacy practice by extending pharmacists’ roles as intersectoral communicators and health educators. The model provides a strategic framework to eliminate systemic health literacy barriers, reduce inequities in access, and encourage appropriate and informed medicines use by positioning pharmacists as intermediaries between educational and healthcare systems [23]. In our sample, the perceptions of collaboration with other healthcare workers were mixed, with agreement and disagreement with the argument present in almost equal measure. Our results correspond with Kamal et al., where only 35.0% of participants reported regular or continuous collaboration with physicians and nurses during their patient-care practice, indicating room for improvement in healthcare collaboration patterns [19]. Other studies have also highlighted this professional collaboration gap, prompting targeted initiatives to strengthen collaborative relationships between healthcare professional staff [24,25]. Nsengimana et al. have identified crucial hallmarks, including inter-professional collaborative care, as the first steps for pharmacists participating in public health initiatives effectively [13].

Jarab et al. suggested that the majority of participants perceived that EPS could be used to generate extra revenue from patients, though, they believed that healthcare providers would not support the introduction of EPS in such fashion [12]; lack of incentives was also noted as a significant barrier by our respondents. Also, consistent with the situation on other LMICs, the absence of clear legal frameworks to define what may be included in EPS (ranging from HPr, medication counseling and reconciliation, immunization or pharmacist prescribing), appropriate training to carry out said services, standardized guidelines and protocols, clear distinctions among public and private pharmacy settings, and finally, reimbursement models to include EPS in government-funded healthcare (i.e., compensation for clinical services), result in under-utilization of pharmacists’ potential. Additionally, clear regulatory interventions are necessary, given that a sizable portion of community pharmacies operate without a qualified pharmacist (staff shortage emerged in our results as well), highlighting a gap between registration and active community, precluding EPS due to a human resource scarcity issue.

Among the EPSs listed, being involved with healthy dietary instructions was most commonly reported, indicating a strong emphasis on preventive health education. According to Elsayed et al., pharmacists expressed favorable opinions about offering patients dietary counseling within the Australian context; they reported already regularly instructing their patients on nutrition within a range of subjects, including dietary intake values, vitamins and minerals [26]. By monitoring special diets (either prescribed by a healthcare professional or otherwise), offering individualized lifestyle counseling, nutrition instruction, and weight control advice, pharmacists could play a critical role in promoting healthy dietary habits, and guidance on when to refer patients to specialists, bridging the gap between nutrition and disease management. In addition, participants reported high levels of involvement in providing education on the practices pertaining to expired or unusable drugs, reflecting a focus on medication safety practices. Hong et al. reported that public knowledge, attitudes, and practices regarding the safe disposal of unused and expired medications has been improved as an outcome of the pharmacist-led educational intervention [27]. By encouraging appropriate disposal methods, pharmacists may aid in reducing the health and environmental risks associated with medicine waste, and they are trusted to offer advice on the risks of using expired products and to organize take-back programs at the pharmacies to limit inappropriate disposal in communal trash or sewage systems. Sendekie et al. also reported on the role of pharmacists in raising awareness of the harmful effects of smoking and alcohol abuse, and the concerns on the community’s growing sedentary lifestyles, which encouraged them to get active [3].

The lowest levels of involvement in EPS activities were reported in the context of family planning counseling; this may be attributed to cultural sensitivities associated with reproductive rights and decisions, limited specific training on the subject and the overall perception that such services fall outside the traditional role of pharmacists. Additionally, pharmacists often have limited involvement in population-based research and campaign activities due to time constraints, lack of institutional support, and insufficient opportunities for engagement in public health initiatives [28]. These findings reflect structural and professional barriers—rather than general disinterest—that restrict pharmacists’ participation in broader public health roles. This was underscored by our current study, where lack of time emerged as one of the most frequently mentioned barriers to engage in EPS, followed closely by lack of resources, limited technology access, or a dedicated room, where counseling could be performed discreetly—findings that concur with earlier reports [12,29]. Other reports from Malaysia [14], the United Kingdom [30], and Jordan [31] likewise found that the absence of a dedicated counseling area was a major barrier to undertake EPS.

The findings of our study should be interpreted with the context of its limitations, such as its cross-sectional design (which precludes the ability of our study to report on causal relationships), convenience sampling (which may introduce selection bias and limit the generalizability of our findings outside of the study’s setting), and the use of a self-reported online survey (which may introduce response and social desirability bias, where participants may over-report their willingness to align with professional ideals). Our sample primarily consisted of fresh graduates and professionals under 5 years of experience; this may limit generalizability to experienced practicing pharmacists, who may have different experiences and attitudes towards EPS introduction. Although the setting of the study (Karachi, Pakistan) is a major population center, pharmacists’ relevance as available healthcare professionals and their experiences may also considerably differ in rural areas, or associated with serving lower-income communities. Thus, additional studies, involving a national representative sample of pharmacists (quantitative) and an in-depth, qualitative analysis (through focus groups or individual interviews) could provide a clearer picture of pharmacists’ perception, and role internalization of involvement in EPS, especially within the context of preventive services and HPr.

## 5. Conclusions

The findings of our study indicate a high-level of willingness by both fresh graduates and practicing pharmacists to provide HPr-focused EPSs in Pakistan, reflecting a positive attitude toward public health interventions and the extension of their professional responsibilities. In fact, many of the participants were actively involved in several patient-centered HPr activities, particularly those related to medication safety, nutrition, and chronic disease counseling, although engagement in broader public health initiatives and family planning services remains comparatively limited and underutilized. Our results suggest that the barriers to engage with EPS are not due to a lack of motivation on the part of the pharmacists, but rather a lack of enabling infrastructure, both in the workplace and the legislative setting. Thus, targeted training and educational programs are recommended to address gaps in pharmacy education and strengthen clinical problem-solving skills; ideally, these skills should be represented within formal undergraduate pharmacy educational programs. Overall, our findings indicate the potential of participants for integrating into broader HPr and public health activities, provided that adequate institutional support and structured training are ensured. In developed countries, pharmacists play important roles in chronic illness management, patient counseling, delivering immunizations, and even prescribing for certain indications, as a part of EPS. However, the current findings reveal similarities with other developing countries, where community pharmacies rarely offer services associated with HPr and prevention, preferring to focus on the conventional tasks of dispensing medications. To bridge the gap between willingness and practice and “real-world” circumstances, the Pharmacy Council of Pakistan must implement standardized protocols and reimbursement models for services beyond dispensing in various pharmacy settings.

## Figures and Tables

**Table 1:** Socio-demographic and professional characteristics of the participants (*N* = 389).

Variable	Category	*n* (%)
Age (Mean ± SD)	—	29.8 ± 4.8 years
Gender	Female	217 (55.8%)
	Male	172 (44.2%)
Pharmacy Practice Setting	Community Pharmacist	45 (11.6%)
	Hospital Pharmacist	69 (17.7%)
	Pharmacy Intern/Fresh Graduate	275 (70.7%)
Organizational Setting	Private sector	269 (69.1%)
	Public sector	120 (30.8%)
Professional Experience	<1 year	196 (50.4%)
	1–5 years	118 (30.3%)
	6–10 years	57 (14.7%)
	Above 10 years	18 (4.6%)
Willing to Provide HPr Services	Maybe	49 (12.6%)
	No	9 (2.3%)
	Yes	331 (85.1%)

HPr: health promotion; SD: standard deviation.

**Table 2:** Pharmacists’ knowledge regarding health promotion and disease prevention (*N* = 389).

	*n* (%)						Statistical Analysis ^a^			
Statement	SD	D	N	A	SA	Gender ^b^	Pharmacy Practice Setting ^c^	Organizational Setting ^d^	Age ^e^	Working Experience ^f^
I have sufficient knowledge to advise patients on HPr and disease prevention	2 (0.5)	49 (12.6)	55 (14.1)	218 (56.0)	65 (16.7)	n.s.	0.036 φ: 0.11	n.s.	<0.001 φ: 0.36	<0.001 φ: 0.38
I can provide health education beyond issues related to medicines.	20 (5.1)	49 (12.6)	60 (15.4)	180 (46.3)	80 (20.6)	0.012 φ: 0.14	n.s.	0.004 φ: 0.29	n.s.	n.s.
I am confident in identifying patients at risk for lifestyle-related illnesses.	15 (3.9)	40 (10.3)	60 (15.4)	200 (51.4)	74 (19.0)	n.s.	n.s.	n.s.	n.s.	0.043 φ: 0.08
I have adequate knowledge to counsel patients on diet and nutrition.	18 (4.6)	75 (19.3)	65 (16.7)	190 (48.8)	41 (10.5)	n.s.	0.017 φ: 0.12	n.s.	0.011 φ: 0.10	0.004 φ: 0.25
I am knowledgeable about preventive measures for common chronic diseases.	22 (5.7)	85 (21.9)	70 (18.0)	175 (45.0)	37 (9.5)	n.s.	n.s.	n.s.	0.035 φ: 0.10	0.013 φ: 0.18

SD: strongly disagree; D: disagree; N: neutral/don’t know; A: agree; SA: strongly agree; HPr: health promotion; ^a^ based on χ^2^-tests or Fisher’s exact tests; ^b^ comparison of males vs. females; ^c^ comparison of community pharmacist vs. hospital pharmacist vs. pharmacy intern/fresh graduate; ^d^ comparison of public vs. private sector; ^e^ comparison of ≤30 years of age vs. >30 years of age; ^f^ comparison of <1 year of experience vs. ≥1 year of experience; n.s.: not significant (*p* > 0.05).

**Table 3:** Pharmacists’ attitudes toward public health activities (*N* = 389).

	*n* (%)					Statistical Analysis ^a^				
Statement	SD	D	N	A	SA	Gender ^b^	Pharmacy Practice Setting ^c^	Organizational Setting ^d^	Age ^e^	Working Experience ^f^
I believe professional curricular training in pharmacy is adequate for providing HPr services.	49 (12.6)	28 (7.2)	51 (13.1)	184 (47.3)	77 (19.8)	n.s.	0.002 φ: 0.37	n.s.	<0.001 φ: 0.42	<0.001 φ: 0.40
I believe pharmacists should actively participate in public health activities.	20 (5.1)	17 (4.4)	32 (8.2)	143 (36.8)	177 (45.5)	<0.001 φ: 0.41	n.s.	n.s.	0.019 φ: 0.18	0.010 φ: 0.17
I feel pharmacists have an important role in public health, along with physicians and nurses.	3 (0.8)	15 (3.9)	32 (8.2)	163 (41.9)	176 (45.2)	n.s.	0.042 φ: 0.09	n.s.	n.s.	n.s.
I believe I have sufficient time to educate patients on health issues.	8 (2.1)	164 (42.2)	63 (16.2)	114 (29.3)	38 (9.8)	n.s.	0.013 φ: 0.20	0.003 φ: 0.31	<0.001 φ: 0.35	<0.001 φ: 0.37
I believe collaboration with other health workers allows pharmacists to perform public health activities more effectively.	36 (9.3)	116 (29.8)	87 (22.4)	111 (28.5)	39 (10.0)	n.s.	n.s.	<0.001 φ: 0.28	n.s.	n.s.
I feel ready to engage in public health activities.	7 (1.8)	28 (7.2)	39 (10.0)	195 (50.1)	118 (30.3)	n.s.	<0.001 φ: 0.37	n.s.	n.s.	n.s.
I believe public health activities can be conducted beyond health centers, including in community pharmacies.	25 (6.4)	131 (33.7)	90 (23.1)	104 (26.7)	39 (10.0)	n.s.	n.s.	n.s.	0.010 φ: 0.10	0.005 φ: 0.19
I believe people will accept and appreciate my involvement in public health activities.	9 (2.3)	70 (18.0)	110 (28.3)	144 (37.0)	56 (14.4)	n.s.	0.016 φ: 0.12	n.s.	0.035 φ: 0.18	0.029 φ: 0.15

SD: strongly disagree; D: disagree; N: neutral/don’t know; A: agree; SA: strongly agree; HPr: health promotion; ^a^ based on χ^2^-tests or Fisher’s exact tests; ^b^ comparison of males vs. females; ^c^ comparison of community pharmacist vs. hospital pharmacist vs. pharmacy intern/fresh graduate; ^d^ comparison of public vs. private sector; ^e^ comparison of ≤30 years of age vs. >30 years of age; ^f^ comparison of <1 year of experience vs. ≥1 year of experience; n.s.: not significant (*p* > 0.05).

**Table 4:** Frequency of self-reported involvement of community and hospital pharmacists in health promotion and public health activities (*n* = 114).

	Never	Rarely	Sometimes	Very Often	Always	Mean ± SD
Activity	*n* (%)					
I provide health education and counseling to encourage smoking cessation.	15 (13.2)	13 (11.4)	17 (14.9)	11 (9.6)	58 (50.9)	3.74 ± 1.42
I provide education to follow special lifestyle recommendations.	7 (6.1)	13 (11.4)	31 (27.2)	17 (14.9)	46 (40.4)	3.72 ± 1.15
I provide health education and counseling to weight management.	1 (0.9)	12 (10.5)	35 (30.7)	20 (17.5)	46 (40.4)	3.88 ± 1.03
I provide health information about oral health and oral hygiene.	4 (3.5)	11 (9.6)	26 (22.8)	32 (28.1)	41 (36)	3.91 ± 1.10
I provide counseling related to the prevention of chronic diseases.	4 (3.5)	5 (4.4)	26 (22.8)	30 (26.3)	49 (43)	4.05 ± 1.01
I provide family planning counseling.	35 (30.7)	25 (21.9)	21 (18.4)	8 (7)	25 (21.9)	2.92 ± 1.36
I provide education about unusable or expired medicine.	3 (2.6)	6 (5.3)	13 (11.4)	17 (14.9)	75 (65.8)	4.36 ± 0.97
I provide education about the use of medical instruments.	10 (8.8)	12 (10.5)	18 (15.8)	25 (21.9)	49 (43)	3.96 ± 1.23
I provide traditional medicine counseling.	10 (8.8)	14 (12.3)	31 (27.2)	21 (18.4)	38 (33.3)	3.77 ± 1.22
I engage in population-based research and initiate public health campaigns.	27 (23.7)	14 (12.3)	35 (30.7)	18 (15.8)	20 (17.5)	3.24 ± 1.33
I provide education to follow a healthy diet.	2 (1.8)	1 (0.9)	17 (14.9)	23 (20.2)	71 (62.3)	4.38 ± 0.89

SD: standard deviation.

## Data Availability

The original contributions presented in this study are included in the article/Supplementary Material. Further inquiries can be directed to the corresponding authors.

## Supplementary Materials

The following supporting information can be downloaded at: https://www.mdpi.com/article/10.3390/pharmacy14030079/s1, Supplementary Material S1: STROBE checklist, Supplementary Materials S2: questionnaire and informed consent forms.
