# Supplementary material for: Willingness, Self-Perceived Barriers, and Practices of Pharmacists Toward Extended Pharmacy Services for Health Promotion: A Cross-Sectional Survey in Karachi, Pakistan
Source: Pharmacy (Basel). 2026 May 28;14(3):79. doi: 10.3390/pharmacy14030079 (PMC13306817; doi:10.3390/pharmacy14030079)
Supplement: Supplementary file 1 [file pharmacy-14-00079-s001.zip › pharmacy-4321851_surveyenglish.pdf]

Pharmacists' willingness, self-perceived barriers and practices towards the provision of Extended Community Pharmacy Services: An Investigative Survey

Informed Consent Form

You are being asked to participate in a research study to explore your willingness, attitude and practices towards extended community pharmacy and public health service provision. There is no risk to participate since we are collecting only your viewpoints. However, the study outcomes would enhance your understanding of your approach toward extended community pharmacy provision in healthcare. Please read this form carefully and ask any questions you may have before agreeing to take part in the study.

What we will ask you to do: If you agree to be in this study, we will ask you to fill out an online questionnaire. This will take about 10-15 minutes to complete.

Risks and benefits:

There is no risk to participate since we are collecting only your viewpoints. There are no benefits or harms to you.

Compensation: You will not get any compensation. Your participation is voluntary.

Your answers will be confidential. The records of this study will be kept confidential. Research records will be kept in a locked file; only the researchers will have access to the records.

Taking part is voluntary: Taking part in this study is completely voluntary. If you decide not to take part, you are free to withdraw at any time.

You will be given a copy of this form for your records.

Statement of Consent: I have read the above information, and agreed to take part. I consent to take part in the study.

Your Signature ______________________ Date ________________________

Name (optional) ____

Signature of person obtaining consent ____________________ Date ___________________

This consent form will be kept by the researcher for at least three years beyond the end of the study.
