# Supplementary material for: Willingness, Self-Perceived Barriers, and Practices of Pharmacists Toward Extended Pharmacy Services for Health Promotion: A Cross-Sectional Survey in Karachi, Pakistan
Source: Pharmacy (Basel). 2026 May 28;14(3):79. doi: 10.3390/pharmacy14030079 (PMC13306817; doi:10.3390/pharmacy14030079)
Supplement: Supplementary file 1 [file pharmacy-14-00079-s001.zip › pharmacy-4321851_surveyform.pdf]

Survey Questionnaire Instrument

Age (in years): ___________ **Gender** Male / Female

Pharmacy practice setting: Community pharmacist / Hospital pharmacist / Pharmacy intern or fresh graduate

Organizational setting: Public sector / Private sector **Highest academic qualification:**____________

Experience: a. <1 year b. 1–5 years c. 6–10 years d. >10 years

Willingness to provide health promotion services: Yes / No / Maybe

**Knowledge**	**Strongly disagree**	**Disagree**	**Neutral /Don't know**	**Agree**	**Strongly Agree**
I have sufficient knowledge to advise patients on health promotion and disease prevention					
I can provide health education beyond issues related to medicines.					
I am confident in identifying patients at risk for lifestyle-related illnesses.					
I have adequate knowledge to counsel patients on diet and nutrition.					
I am knowledgeable about preventive measures for common chronic diseases.					
**Perception**	**Strongly disagree**	**Disagree**	**Neutral /Don't know**	**Agree**	**Strongly Agree**
I believe professional curricular training in pharmacy is adequate for providing health promotion services.					
I believe pharmacists should actively participate in public health activities.					
I feel pharmacists have an important role in public health, along with physicians and nurses.					
I believe I have sufficient time to educate patients on health issues.					
I believe collaboration with other health workers allows pharmacists to perform public health activities more effectively.					
I feel ready to engage in public health activities.					
I believe public health activities can be conducted beyond health centers, including in community pharmacies.					
I believe people will accept and appreciate my involvement in public health activities.					

Your involvement in Public Health Activities

Activity	Never	Rarely	Sometimes	Very Often	Always	Not applicable
I provide health education and counselling to encourage smoking cessation.						
I provide education to follow special lifestyle recommendations.						
I provide health education and counselling to weight management.						
I provide health information about oral health and oral hygiene.						
I provide counseling related to the prevention of chronic diseases.						
I provide family planning counseling.						
I provide education about unusable or expired medicine.						
I provide education about the use of medical instruments.						
I provide traditional medicine counseling.						
I engage in population-based research and initiate public health campaigns.						
I provide education to follow a healthy diet.						

Perceived Barriers (Select all that apply)

a. Lack of time
b. Lack of skills
c. Lack of resources
d. Limited access to technology
e. Lack of protocols
f. Low engagement
g. Lack of incentives
h. Lack of patient interest
i. Lack of privacy for counselling
j. Financial constraints
k. Staff shortage
l. Other ____________________
