# Supplementary material for: Willingness, Self-Perceived Barriers, and Practices of Pharmacists Toward Extended Pharmacy Services for Health Promotion: A Cross-Sectional Survey in Karachi, Pakistan
Source: Pharmacy (Basel). 2026 May 28;14(3):79. doi: 10.3390/pharmacy14030079 (PMC13306817; doi:10.3390/pharmacy14030079)
Supplement: Supplementary file 1 [file pharmacy-14-00079-s001.zip › pharmacy-4321851_surveyurdu.pdf]

کنسینٹ فارم

Pharmacists' willingness, self-perceived barriers and practices towards the provision of Extended Community Pharmacy Services: An Investigative Survey

میں رضاکارانہ طور پر اس تحقیق میں حصہ لینے پر راضی ہوں۔ تحقیق ، مطبوعات ، ڈیٹا شیئرنگ اور میرے ڈیٹا کے استعمال کی وضاحت کی گئی ہے۔ میں سمجھتا ہوں کہ میں کسی بھی وقت وجوہات بتائے بغیر تحقیق سے دستبرداری لے سکتا ہوں اور دستبرداری کرنے پر مجھ سے پوچھ گچھ نہیں کی جائے گی کہ میں کیوں پیچھے ہٹ گیا ہوں۔
مجھے اس تحقیق اور میری شرکت کے بارے میں سوالات پوچھنے کا موقع فراہم کیا گیا ہے۔ میں رضامندی کے ساتھ اس تحقیق میں حصہ لینے اوراس فارم پر دستخط کرنے پر راضی ہوں ۔

نام | دستخط | تاریخ
